# Hypertrophic Cardiomyopathy as a Key Feature of MRAS‐Related Noonan Syndrome: New Case and Comprehensive Literature Review

**DOI:** 10.1002/pd.70134

**Published:** 2026-03-22

**Authors:** Romain Martineau, Constance Wells, Florent Fuchs, Sophie Collardeau‐Frachon, Valentin Ruault, Sophie Colomb, Jean‐Michel Faure, Caroline Bartholmot, Marie Vincenti, Benjamin Ganne, Marjolaine Willems

**Affiliations:** ^1^ Resident in Gynecology Obstetrics and Fetopathology Montpellier University Montpellier France; ^2^ Medical Genetics Department University Hospital of Montpellier Montpellier University Montpellier France; ^3^ Department of Obstetrics and Gynecology University Hospital of Montpellier Montpellier University Montpellier France; ^4^ Inserm CESP Center for Research in Epidemiology and Population Health Reproduction and Child Development Villejuif France; ^5^ DESBREST Institute of Epidemiology and Public Health (IDESP) University of Montpellier INSERM Montpellier France; ^6^ Department of Pathology Hospices Civils de Lyon & Université Claude Bernard Lyon 1 Lyon France; ^7^ Montpellier Criminal Law and Forensic Sciences Team (EDPFM) University of Montpellier Montpellier France; ^8^ Department of Forensic Medicine Emergency Department Montpellier University Hospital Center Montpellier France; ^9^ Department of Pediatric and Congenital Cardiology M3C Regional Reference Centre Montpellier University Hospital Montpellier France; ^10^ PhyMedExp INSERM CNRS University of Montpellier Montpellier France; ^11^ Molecular Genetics and Cytogenomics Department University Hospital of Montpellier Montpellier University Montpellier France; ^12^ Institute for Neurosciences of Montpellier University Montpellier INSERM Montpellier France

**Keywords:** fetal pathology, hypertrophic cardiomyopathy, MRAS, noonan syndrome, prenatal, RASopathies

## Abstract

Noonan syndrome (NS) is a rare multisystemic condition among the RASopathy group, characterized by a broad phenotypic spectrum and genetic variability. It results from pathogenic variants in genes regulating the RAS/MAPK pathway, affecting cell proliferation and differentiation. While the *PTPN11* gene accounts for approximately 50% of cases, other genes, including *MRAS*, have been implicated. NS presents with features such as facial dysmorphism, short stature, and cardiac anomalies. Hypertrophic cardiomyopathy (HCM) is a major contributor to mortality, with specific variants conferring higher risk. This article includes a review of the literature on NS with pathogenic *MRAS* variants and describes an eighth case, the first documented with early and severe antenatal manifestations. The fetus exhibited increased nuchal translucency, agenesis of the ductus venosus, pulmonary lymphangiectasia, and complex hepatic vascular anomalies. A cesarean section was performed at 33 weeks' gestation due to worsening fetal pleural effusions and maternal intolerance to polyhydramnios. Despite intensive postnatal care, the newborn died from refractory shock and multi‐organ failure. Histopathology revealed HCM, obliterative portal venopathy and lymphangiectasia, consistent with NS pathology. These findings suggest that pathogenic *MRAS* variants confer a high risk of severe HCM (100% of cases). Moreover, emerging targeted therapies, such as MEK inhibitors, offer potential for treatment.

## Summary

- What's already known about this topic?
  ◦ Noonan syndrome (NS) is a RASopathy linked mainly to pathogenic variation of *PTPN11* gene.
  ◦ *MRAS*‐related NS is rare (7 cases), all with hypertrophic cardiomyopathy (HCM), but its phenotype is not fully defined.
- What does this study add?
  ◦ This is the first case with detailed prenatal imaging and pathology and the first review of *MRAS*‐related NS.
  ◦ It confirms the *MRAS*‐HCM link and expands the phenotypic spectrum.

## 1 Introduction

Noonan syndrome is a rare and multisystemic disorder with highly variable and often subtle manifestations, as reflected by a median age at diagnosis of 4 years [1]. The birth prevalence of Noonan syndrome is estimated to be between 1 in 1000 and 1 in 2500 births, making it the most common RASopathy [2]. RASopathy is a group of disorders primarily affecting the nervous system, heart, skin and craniofacial development. These conditions result from pathogenic variants affecting genes encoding components of the RAS/MAPK signaling pathway, a phosphorylation cascade involved in regulating cell proliferation, differentiation, and survival [2, 3].

Noonan syndrome is characterized by significant genetic variability: in approximately 50% of cases, it is caused by pathogenic variants in the *PTPN11* gene (12q24.13), but variants in other genes have been identified (*SOS1* (2p22.1), *RAF1* (3p25.2), *RIT1* (1q22), *LZTR1* (22q11.21)). In most cases, Noonan syndrome is inherited in an autosomal dominant manner [1, 3], with heterozygous activating variants resulting in hyperactivation of the RAS/MAPK pathway [4]. However, autosomal recessive forms associated with biallelic pathogenic variants in *LZTR1* have also been described [2, 5].

Prenatal signs of Noonan syndrome as well as findings in fetal pathology are not specific, including increased nuchal translucency, cystic hygroma, serosal effusions or hydrops (consequences of lymphatic drainage disorders), polyhydramnios, or dysmorphism (low‐set ears, frontal bossing, downward‐slanting palpebral fissures, etc.). Hypertrophic cardiomyopathy (HCM), congenital cardiac defects, and neurological abnormalities may also be present [6, 7]. Generally, ultrasound findings suggest the possibility of Noonan syndrome, prompting either prenatal molecular diagnosis or postnatal confirmation based on the couple's preference.

After birth, the main clinical features of this syndrome can include facial dysmorphism, a short and/or webbed neck, short stature, chest and/or spinal deformities, cardiac anomalies, cryptorchidism, hematological disorders, varying learning difficulties and an increased predisposition to cancer [2, 4]. In the neonatal period, Noonan syndrome most often manifests with feeding difficulties and growth restriction [1].

Moreover, the expressivity of this syndrome is highly variable, ranging from minimally symptomatic adults to severely affected neonates with premature mortality [1]. The clinical presentation also varies according to the gene involved, and certain conditions have historically been described as “Noonan‐like” syndromes (e.g., juvenile myelomonocytic leukemia, Noonan‐like disorder with loose anagen hair) [1]. Early diagnosis is necessary to ensure individualized and appropriate management approach [8]. Although no consistent genotype‐phenotype correlation has been clearly established in Noonan syndrome, pathogenic variants in specific genes are more frequently associated with hypertrophic HCM [4, 9].

In 2017, a new RAS family gene, *MRAS*, has been implicated as a cause of Noonan syndrome. In the literature, seven unrelated patients have been reported, all presenting with severe HCM [10, 11, 12, 13, 14], a cardiac phenotype that appears more severe than in other pathogenic variants. These cases also exhibited features consistent with other Noonan syndromes, including short stature and facial dysmorphism. Notably, no antenatal cases have been described with *MRAS* variant.

Here, we report the eighth case of Noonan syndrome caused by a pathogenic *MRAS* variant, the first with severe antenatal manifestations. This patient also exhibited some clinical and histological features compared with previously described cases, which will be detailed in the following sections of this article. We also review the literature on *MRAS*‐related Noonan syndrome to better delineate its phenotypic spectrum.

## 2 Materials and Methods

A literature review was performed using *PubMed* to identify reported cases of *MRAS*‐related Noonan syndrome. The data are summarized in Table 1. The research was conducted in February 2025 by a single reviewer using the *MeSH* (Medical Subject Heading) terms “Noonan” and “MRAS”. Articles were included if they described clinical phenotypes in human cases with confirmed pathogenic *MRAS* variants, whether diagnosed prenatally, neonatally, in childhood, or in adulthood. Non‐English or French articles (but ultimately all the articles found were written in English), review articles without primary case data, and animal studies were excluded.

**TABLE 1:** Summary of the literature review of cases of Noonan syndrome caused by pathogenic variation in the *MRAS* gene.

Reference	Age at diagnostis	Antenatal features	Sex	Origin	Cardiac findings	Evolution	Other signs	*MRAS* variants
Higgins et al, 2017 [10]	15 years old	NS	F	Europe	Hypertrophic cardiomyopathy	Surgery (myectomy, 8 years old)	Short stature, facial dysmorphisms, developmental delay and cognitive impairment	NM_012219.4:c.68 G > T; p.Gly23Val De novo
Higgins et al, 2017 [10]	6 years old	NS	F	Europe	Severe hypertrophic cardiomyopathy, atrial septal defect, valvar pulmonic stenosis	NS	Facial dysmorphisms, pectus excavatum, deep palmar creases, moderate developmental/intellectual compromises	NM_012219.4:c.203 C > T; p.Thr68Ile De novo
Suzuki et al, 2019 [12]	1 year and 3 months	NS	M	Japan	Hypertrophic cardiomyopathy	Cardiac arrest in the newborn period Evolution over 1 year and 3 months: NS	Short stature, poor weight gain, relative macrocephaly, facial dysmorphisms, hearing loss, developmental delay	NM_012219.4:c.212 A > G; p.Gln71Arg De novo
Motta et al, 2020 [11]	2 years and 3 months	“Uneventful pregnancy”	M	North Africa	Hypertrophic cardiomyopathy, atrial septal defect	Concentric hypertrophic cardiomyopathy at 3 years	Short stature, facial dysmorphisms, pectus excavatum, global developmental delay	NM_012219.4:c.203 C > T; p.Thr68Ile De novo
Motta et al, 2020 [11]	Newborn	“Uneventful pregnancy”	F	Europe	Hypertrophic cardiomyopathy (marked biventricular hypertrophy)	**Death** (2 months)	Facial dysmorphisms, short neck	NM_012219.4:c.67 G > C; p.Gly23Arg De novo
Pires et al, 2021 [13]	Newborn	“No malformations detected by fetal Ultrasound”	M	Brasil	Hypertrophic cardiomyopathy, atrial septal, defect, left ventricule apic aneveysm	Surgery (myectomy, 1 month) **Death** (1 day post‐surgery, arrythmia)	Facial dysmorphisms, widely spaced nipples, deep plantar creases	NM_012219.4:c.203 C > T; p.Thr68Ile De novo
Priolo et al, 2023 [14]	40 years old	NS	M	NS	Hypertrophic cardiomyopathy (mild hypertrophic left ventriculum), interatrial septal aneurism, mitral valve prolapse	Stable No surgery	Facial dysmorphism, psychomotor delay, epilepsy (complex partial seizures), psychiatric symptoms, short neck scoliosis, dorsal kyphosis, pectus carenatum	NM_012219.4:c.203C > T p.Thr68Ile
Present study	Newborn	Increased nuchal translucency, agenesis of the ductus venosus, cystic hepatic lesion, polyhydramnios	F	Europe	Hypertrophic cardiomyopathy, atrial septal defect, aneurysmal pulmonary arterial trunk	**Death** (at 5 days)	Facial dysmorphism, anasarca, congenital portosystemic shunt, histological liver and lung lesions, bile cyst	NM_012219.4:c.68G > T, p.Gly23Val De novo

Abbreviations: F, female; M, male; NS, Not Specified.

This search retrieved 24 publications from *PubMed*, from which seven cases [10, 11, 12, 13, 14] with *MRAS* pathogenic variants were identified after screening titles, keywords and abstracts. These included neonatal, pediatric, and adult presentations. A similar search of *Google Scholar* identified no additional cases. Among these, only two cases were indexed in *OMIM* (entry #608435) [10, 12] and there were no additional cases. The *Gene2Phenotype* database lists five distinct cases: the two from *Higgins* et al. [10], one from *Suzuki* et al. [12], and two from *Motta* et al. [11] (i.e., no additional cases).

On *ClinVar*, combining the terms “Noonan” and “*MRAS*” yielded seven variants: three classified as benign, one of uncertain significance, and three as likely pathogenic. However, none of these entries were associated with detailed clinical phenotype data. The three variants classified as likely pathogenic included: NM_012219.4:c.68G > T (p.Gly23Val), which was also found in one of the cases reported by *Higgins* et al. [10] and in our study; NM_012219.4:c.203C > T (p.Thr68Ile), found in four of our cases [10, 11, 13, 14]; and NM_012219.4:c.212A > G (p.Gln71Arg), reported in one of our cases [12]. Thus, the *ClinVar* database, which lists human genetic variations, contains seven of the eight cases in our study and did not allow us to identify any other cases. Therefore, the only pathogenic variant not referenced in *ClinVar* was NM_012219.4:c.67G > C (p.Gly23Arg), as reported by *Motta* et al. [11].

Finally, on the *Franklin* platform by *QIAGEN* (genoox.com), seven cases of pathogenic variants are referenced, all corresponding to missense variants, consistent with our literature review: the seven cases on this platform did indeed correspond to the seven cases already identified.

Given the very small number of documented cases and the limited availability of secondary sources, a targeted narrative review was considered more appropriate than a systematic review approach based on the PRISMA methodology. Moreover, the data were verified by a second reviewer. The last search was conducted on May 13, 2025, and no additional cases were found thereafter.

## 3 Result

### 3.1 Case Presentation

**FIGURE 1:**
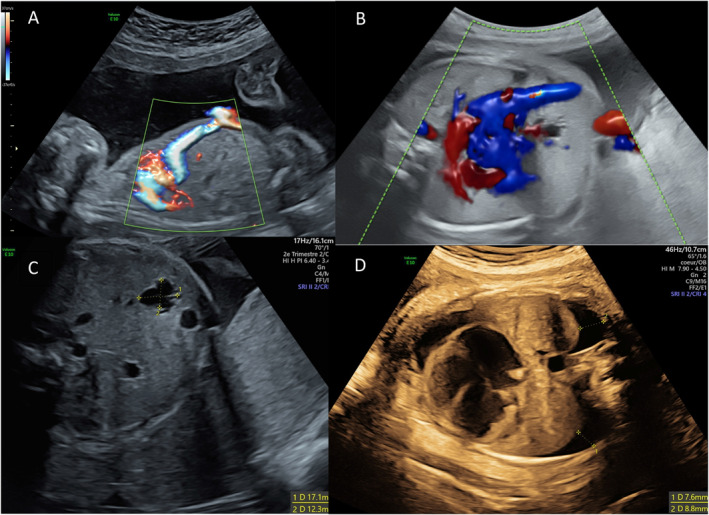
Obstetrical ultrasound images of interest obtained transabdominally at different gestational ages. (A) Right parasagittal view centered on the thorax and abdomen at 22 + 1 week' gestation (WG)—Color Doppler mode—Anomalous course of the umbilical vein draining directly into the right atrium without passing through the liver. Agenesis of the ductus venosus. (B) Axial view centered on the heart at 27 + 3 WG—Umbilical vein draining directly into the right atrium. (C) Axial view centered on the liver at 32 + 1 WG—Breech presentation, back to the right—Single heterogeneous cystic hepatic image in segment IV. (D) Axial view at 32 + 5 WG—Breech presentation, back to the left—Bilateral pleural effusions with rightward cardiac deviation.

This was the second child of a non‐consanguineous couple with an unremarkable medical history. Increased nuchal translucency was detected on first‐trimester ultrasound. Invasive testing (chorionic villus sampling) revealed a normal karyotype (46,XX) and normal chromosomal microarray analysis (CMA). The ultrasound at 17 + 2 weeks' gestation (WG) showed agenesis of the ductus venosus without an intrahepatic course of the umbilical vein, which drained directly into the coronary sinus. The cardiac apex was displaced to the right without evidence of cardiopathy (Figure 1A‐B). Subsequent ultrasounds remained unchanged until 32 + 1 WG, when a single cystic hepatic lesion in segment IV, consistent with a probable ciliated cyst (Figure 1C), increased pulmonary blood flow, and left pleural effusions were identified. The effusion became bilateral at 32 + 5 WG (Figure 1D). Because of acute polyhydramnios caused significant maternal discomfort and worsened fetal pleural effusions, an emergency cesarean delivery was performed at 33 WG. The female infant weighed 2400 g (+1.3 Standard Deviation SD) and measured 43 cm (−0.3 SD). The Apgar scores were 1, 0, and 3 at 1, 5, and 10 min, respectively. Pediatric resuscitation required orotracheal intubation and bilateral pleural drainage. Postnatal ultrasounds confirmed the pleural effusions, rightward heart deviation with right ventricular hypertrophy, subsystemic pulmonary hypertension, and a segment IVa lesion with agenesis of the portal system. The infant rapidly developed heart failure, leading to refractory shock, multi‐organ failure, coagulopathy, and death on day 5 of life.

**FIGURE 2:**
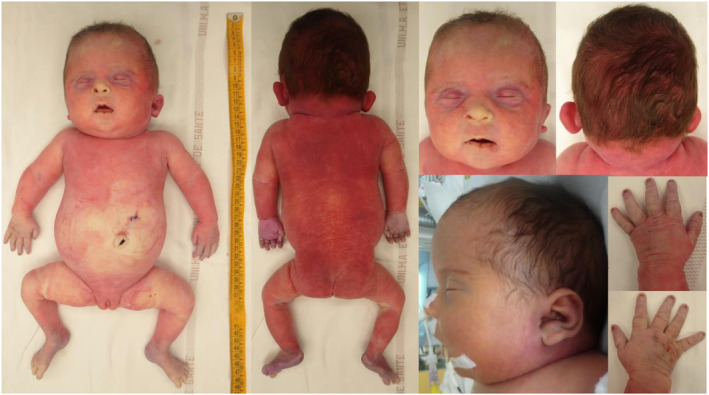
Photographs of the whole body (frontal and dorsal views); facial and neck photographs; child's profile; dorsal view of the hands.

At clinical examination, the weight was 2726 g (> 95th percentile for gestational age) in a context of hydrops fetalis, with diffuse erythema and fine reticulations, especially over the lumbar region and buttocks. The head circumference was 32 cm (75–90th percentile). Craniofacial examination (Figure 2) revealed a high forehead, a broad‐based nose with a short and wide columella, a small mouth with a thin upper lip and an interposed tongue. Brachycephaly with plagiocephaly and a short neck were observed. The ears were low‐set with folded lobes and edema. The infant exhibited rhizomelia and toenail hypoplasia (Figure 2). The abdomen was broad, with slight umbilical deviation. The anus was anteriorly displaced.

**FIGURE 3:**
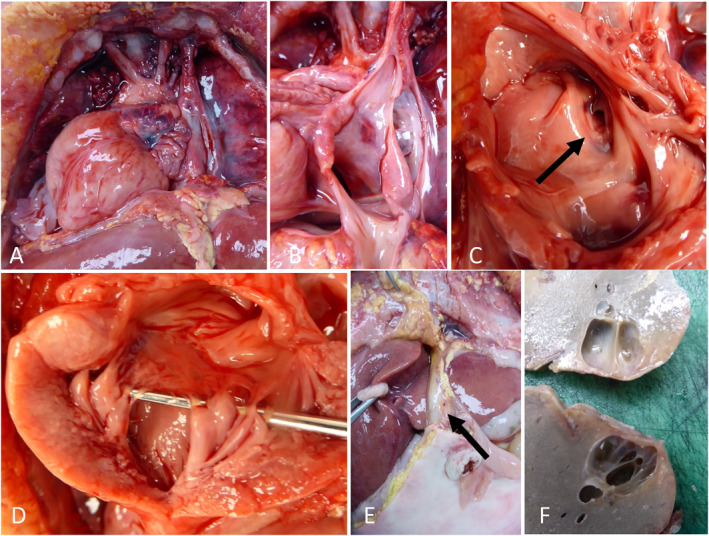
Autopsy photographs. (A–D) Cardiovascular dissection. (A) Frontal external view of the heart showing cardiomegaly with rightward cardiac apex deviation, aneurysmal pulmonary artery trunk, and presence of a left superior vena cava. Right superior vena cava not visualized. (B) Left superior vena cava draining into the coronary sinus along with the umbilical vein. (C) Atrial septal defect (arrowhead). (D) Thickened mitral valve and left ventricular papillary muscles. (E) Umbilical vein draining directly into the coronary sinus bypassing the liver. (F) Biliary cyst after fixation in 4% formaldehyde.

At autopsy, there were bilateral pleural effusions, dense lungs with yellowish pleural adhesions and bifid xiphoid. Cardiovascular anomalies (Figure 3A–D) included marked cardiomegaly, rightward cardiac axis deviation and a tortuous aortic arch with intimal folds and pulmonary trunk aneurysm. The interventricular septum was thickened with thickening of the mitral valve apparatus and right ventricular papillary muscles. Multiple atrial septal defects were present. A persistent left superior vena cava drained into the coronary sinus. The umbilical vein emptied directly into the right atrium from the umbilicus without an intrahepatic course (Figure 3E). In the abdominal and pelvic regions, there were ascites and diaphragmatic dehiscence without hernia. The liver was dysmorphic and contained a cyst of approximately 1 cm in segment IV (Figure 3F); the gallbladder was deviated and the common bile duct was short. The portal vein was atretic proximal to the liver (prehepatic atresia). The left hepatic artery originated from the celiac trunk, whereas the right hepatic artery originated from the superior mesenteric artery. Ductus venosus agenesis was confirmed. There was mild splenomegaly.

Placental histology revealed prominent villous edema consistent with hydrop fetalis; an acute funiculitis was also observed. In contrast to our previous case of Noonan syndrome [15], no evidence of heterotopic tissue (including hepatic or adrenal), was observed.

Pulmonary lymphangiectasia was identified on histology, characterized by dilated D2‐40 positive vessels lacking a muscular medial layer and without features of alveolocapillary dysplasia (Supporting Information S1: Appendix 1A‐C). In the bronchovascular pedicles, some dilated structures corresponded to the vasa vasorum, as described in pulmonary arterial hypertension or systemic to pulmonary shunts (Supporting Information S1: Appendix 1A). The hepatic cyst was multiloculated and lined by a single layer of endothelium‐like squamous epithelium expressing CK7, suggesting a biliary origin (Supporting Information S1: Appendix 1D‐E). The hepatic parenchyma showed obliterative portal venopathy with fibro‐edematous portal tract widening and porto‐portal bridging (incomplete septal fibrosis), hypoplastic or absent portal vein branches, and increased hepatic arterial branches (Supporting Information S1: Appendix 1F‐G). The sinusoids were dilated with a slight collapse of the hepatocyte trabeculae and some signs of hepatocellular distress characterized by microvacuolar hypoxia. The central lobular veins were sometimes dilated with thickened walls, possibly linked to right heart failure. Mild hemosiderosis on Perls' stain (Prussian blue) was consistent with cardiac liver disease. The remainder of the histological examination showed right‐predominant myocardial hypertrophy and lymphoid depletion in the thymus and spleen, likely secondary to chronic hypoxia, with congested red pulp consistent with splenomegaly. Other organs and neuropathological examination were unremarkable.

Trio exome sequencing identified a de novo pathogenic *MRAS* variant, NM_012219.4:c.68G > T (p.Gly23Val), previously reported and classified as pathogenic (“Class 5”: PS3, PS4,PS2, PM6, PM2, PS1,PM5, PP3 [16]). Technical details are provided in Supporting Information S2: Appendix 2.

### 3.2 Literature Review

The literature review identified seven cases (Table 1). This review shows a 1:1 sex ratio (four males, four females), with half of the subjects being of European descent. Based on available follow‐up data, the mortality rate reached 37.5% (three of eight individuals). Three cases (including this case) were diagnosed in the neonatal period; for the remaining five, the median age at diagnosis was 12.9 years, with the oldest diagnosed at 40 years. None of the seven cases published prior to ours included a prenatal description.

Recurrent features among these cases included facial dysmorphism, short stature, and mostly hypertrophic cardiomyopathy for all, which weighs on the prognosis. It should be noted that a developmental delay is described for all surviving patients.

On the genetic level, the eight cases of Noonan syndrome presented with missense variants in *MRAS* codons p.Gly23, p.Thr68, and p.Gln71. All subjects carried de novo pathogenic variants, with no inherited cases identified. A synthesis of this review is provided in Table 1.

## 4 Discussion

This literature review demonstrates that, to date, all reported cases of Noonan syndrome caused by pathogenic *MRAS* variants have presented with hypertrophic cardiomyopathy and characteristic facial dysmorphism. Mortality appears high (3 out of 8 cases). Our case provides a detailed prenatal and histopathological description and highlights several atypical features, including hepatic and pulmonary vascular anomalies, hepatic cysts, and pulmonary trunk aneurysms.

In our case, the pathogenic *MRAS* variant explains the phenotype observed, in particular increased nuchal translucency, dysmorphic features, cardiomyopathy, ductus venosus agenesis and pulmonary lymphangiectasia. The hepatic vascular abnormalities (combining ductus venosus agenesis and portal system hypoplasia, resembling Abernethy type II malformation and obliterative portal venopathy), complicated by pulmonary vascular abnormalities (hepato‐pulmonary syndrome and/or pulmonary arterial hypertension) may represent part of Noonan syndrome spectrum. From a pathophysiological perspective, the pulmonary vascular changes may be secondary to hepatopulmonary syndrome in the setting of hepatic vascular anomalies. After a multidisciplinary expert review, these hepatic and portal abnormalities were considered manifestations of *MRAS* associated with Noonan syndrome.

More generally, the most common antenatal ultrasound findings of Noonan syndrome include cystic hygroma or increased nuchal translucency in the first trimester, excess fluid or polyhydramnios, pleural or pericardial effusions, and cardiac and renal malformations [1]. All of these features, except renal malformations, were present in our case, underscoring the nonspecific nature of the antenatal findings with respect to *MRAS* variants. Moreover, among previously reported *MRAS*‐cases, short stature, thoracic anomalies, and facial dysmorphism were frequent, and were also present in our patient. Early onset and rapidly unfavorable progression were also observed in other children with *MRAS* pathogenic variants; however, this is the first documented case with detailed prenatal findings and a rapidly fatal outcome. Overall, published cases suggest a severe phenotype and poor prognosis (Table 1), although the number of reported patients remains limited.

Due to the small number of cases, the phenotype of *MRAS*‐related Noonan syndrome remains to be fully defined. Nevertheless, the cardiac phenotype, particularly hypertrophic cardiomyopathy, has been consistently observed across all cases. This contrasts with the overall prevalence of HCM (∼20%) in Noonan syndrome [4, 10, 11, 12, 13, 14]. In RASopathies, the HCM prevalence varies according to the gene involved: approximately 70% for *HRAS* pathogenic variants or 25% with *KRAS* [17]; *NRAS*‐related Noonan syndrome appears to have an intermediate HCM prevalence between *HRAS* and *KRAS* cases [18]. Thus, *MRAS* pathogenic variants appear to confer one of the highest risks for HCM within the RAS family, with a 100% prevalence among reported cases in this review. Moreover, cardiomyopathy is a key factor in increased mortality [1]. Taken together, these findings suggest that *MRAS* variants may represent high‐risk factors for the development of severe hypertrophic cardiomyopathy, potentially associated with poor prognosis.

*Higgins* et al. [19] demonstrated the crucial role of *MRAS* in cardiomyocytes using induced pluripotent stem cells (iPSCs) derived from a Noonan syndrome patient with HCM and an *MRAS* p.Gly23Val variant. Their results demonstrated that this pathogenic variant was both necessary and sufficient to induce myocardial hypertrophy through hyperactivation of the RAS/MAPK signaling pathway. Furthermore, pharmacological inhibition of this pathway significantly reduced hypertrophy in iPSC‐derived cardiomyocytes, highlighting the therapeutic potential of this approach. Indeed, the administration of selective MEK inhibitors (e.g., Trametinib) has shown beneficial effects with regression of myocardial hypertrophy in two infants with severe HCM carrying pathogenic variants in *RIT1* [20]. These targeted therapies, which specifically act on the RAS/MAPK signaling pathway, could thus represent a promising therapeutic approach for managing severe forms of HCM associated with RASopathies.

In our case, the newborn suffered from cardiomegaly with a marked hypertrophy of the interventricular septum and diffuse myocardial hypertrophy, as well as multiple atrial septal defects. These observations have already been reported in previous cases (Table 1). However, an atypical cardiac anomaly was observed: the pulmonary arterial trunk was aneurysmal above the right ventricle and up to the bifurcation. To date, the literature reports only isolated cases of ventricular aneurysms, including an apical aneurysm of the left ventricle described by *Pires* et al. [13] and a ventricular aneurysm reported by *Tozzi* et al. [21].

To our knowledge, there are no reports of biliary cysts in association with Noonan syndrome. However, two articles describe Abernethy malformations associated with Caroli disease [22, 23]. Nevertheless, after reviewing the exome sequencing data, no pathogenic variants were identified in the *PKHD1* gene (associated with Caroli disease) and there was also no suggestive family history.

Furthermore, unlike classical *RAS* oncogenes (*HRAS, NRAS, KRAS*), somatic variants in *MRAS* are rarely observed in cancers. However, the germline *MRAS* p.Gln71Arg variant, reported by *Suzuki* et al. [12], has been identified in several types of tumors.

## 5 Conclusion

This report describes one of the few documented cases of Noonan syndrome caused by a pathogenic *MRAS* variant, and to our knowledge, the only case with antenatal manifestations severe enough to cause early neonatal death. In addition to the cardiac involvement commonly associated with *MRAS*‐related disease, this case is notable for the coexistence of hepatic and pulmonary vascular anomalies as well as a biliary cyst, suggesting a broader systemic impact than previously recognized. The combination of prenatal onset, multi‐organ involvement, and rapidly fatal course highlights the importance of considering *MRAS* variants in the differential diagnosis of severe fetal presentations of RASopathies.

Our literature review further indicates that patients with *MRAS*‐related Noonan syndrome have a particularly poor cardiac prognosis, with hypertrophic cardiomyopathy observed in all cases and high mortality rates, while survivors may experience neurodevelopmental impairments. The accumulation of additional cases, including detailed prenatal observations, will be essential to more precisely define the phenotypic spectrum, clarify genotype–phenotype correlations, and guide prognostic counseling and management strategies.

## Funding

The authors have nothing to report.

## Ethics Statement

The authors have nothing to report.

## Consent

Written informed consent for publication of clinical details and clinical images (ultrasounds, autopsy, histology) was obtained from the parents of the patient.

## Conflicts of Interest

The authors declare no conflicts of interest.

## Supporting information


Supporting Information S1

Supporting Information S2

## Data Availability

The data that support the findings of this study are available from the corresponding author upon reasonable request.
