# Supplementary material for: Hypertrophic Cardiomyopathy as a Key Feature of MRAS‐Related Noonan Syndrome: New Case and Comprehensive Literature Review
Source: Prenat Diagn. 2026 Mar 22;46(4):581–8. doi: 10.1002/pd.70134 (PMC13070222; doi:10.1002/pd.70134)
Supplement: Supplementary file 1 — Supporting Information S1 [file PD-46-581-s001.docx]

**Appendix 1**

Histological sections stained with Hematoxylin and Eosin (A, B, D, F, G) and immunohistochemistry (C, E).

*
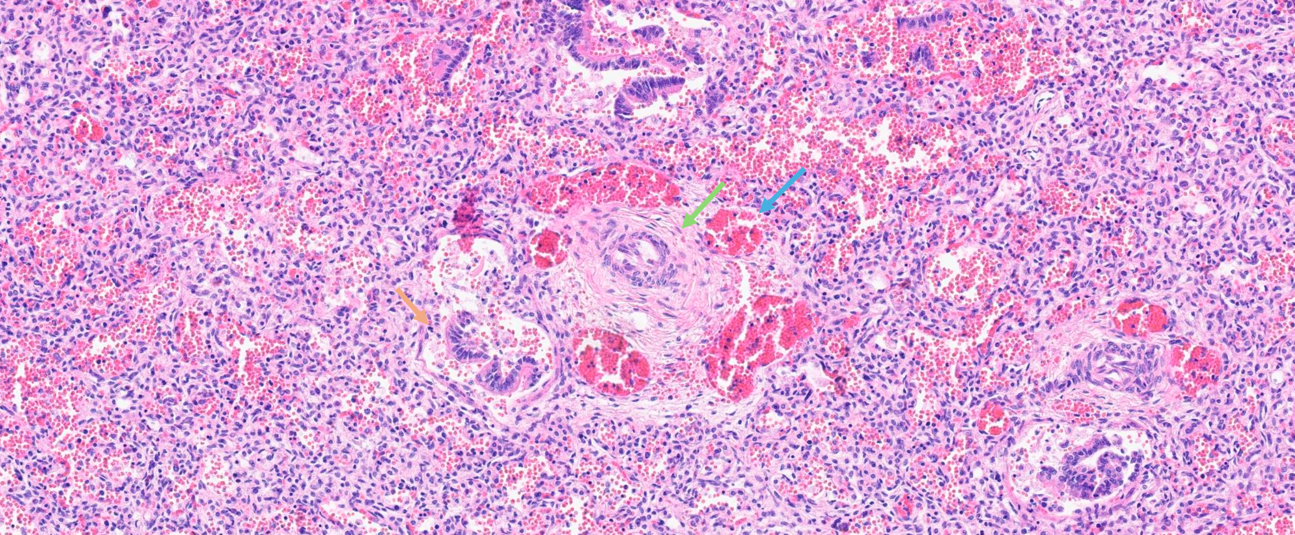
*

**A.** Fetal lung section centered on a bronchovascular pedicle (magnification ×12).

**
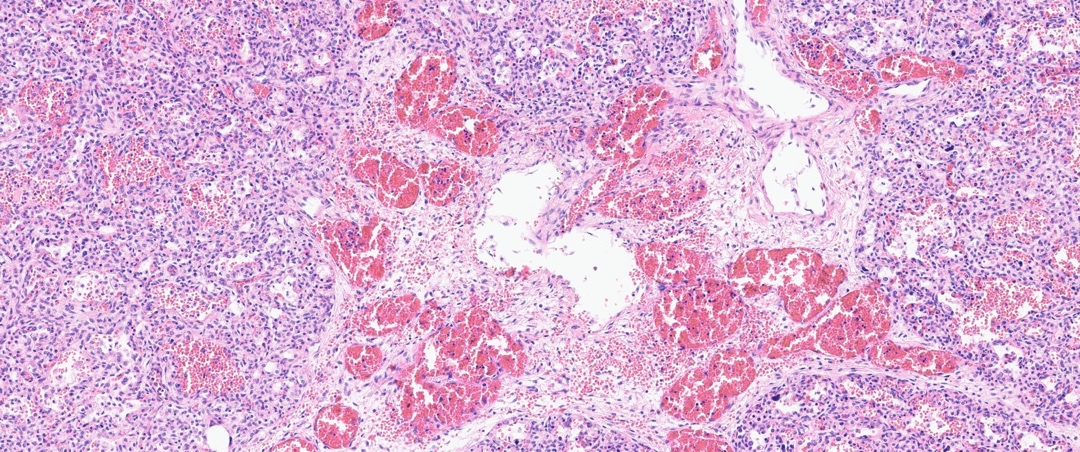

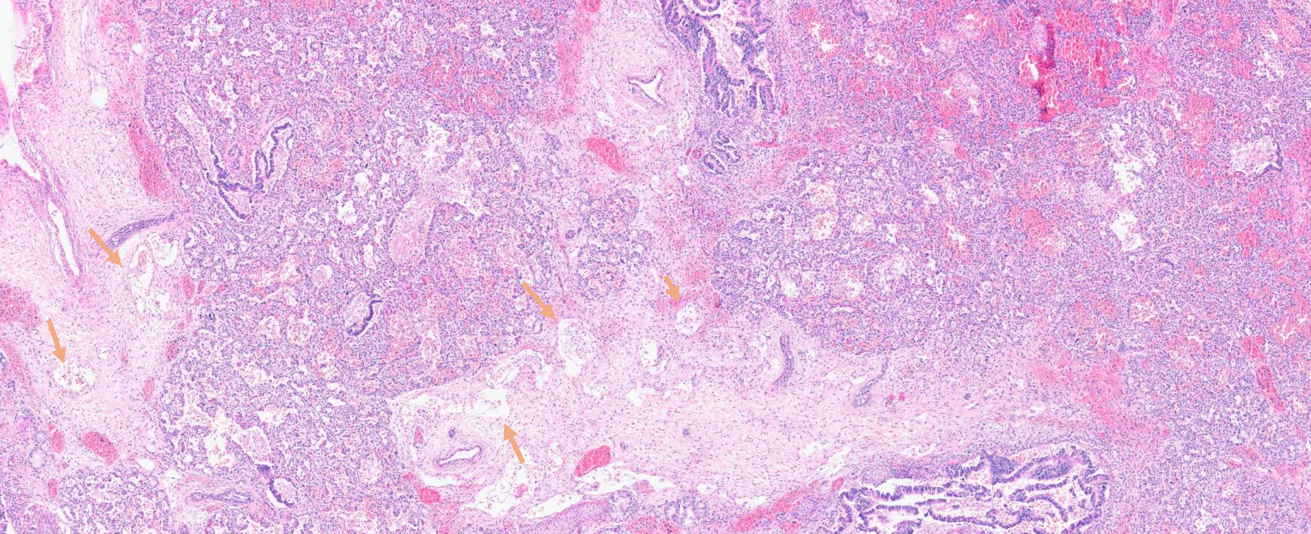
**Blue arrow : dilated vasa vasorum. Green arrow: pulmonary artery branch. Orange arrow: bronchus. No evidence of alveolocapillary dysplasia (pulmonary veins are not located in the arterial adventitia, and no capillary rarefaction is seen in alveolar septa).

**B**. Fetal lung section (×3).

Orange arrows : numerous lymphangiectasias.

On the right : lymphangiectasias on magnification 6.7.


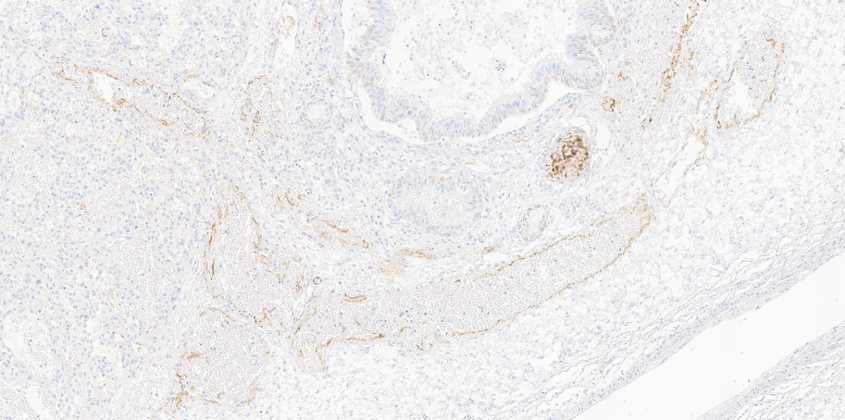


**C.** Immunohistochemistry showing D2-40 expression by endothelium lacking a media layer, consistent with lymphatic vessels.

**
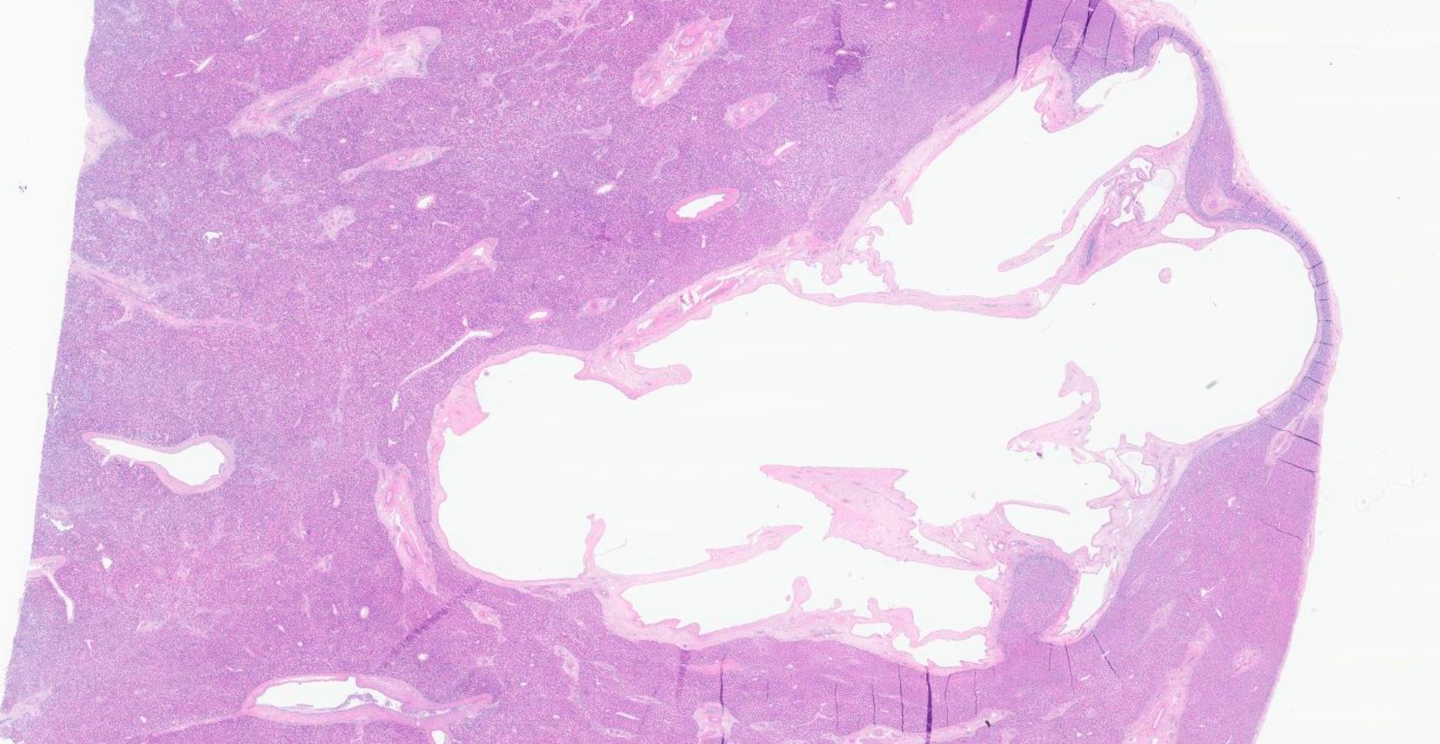
**


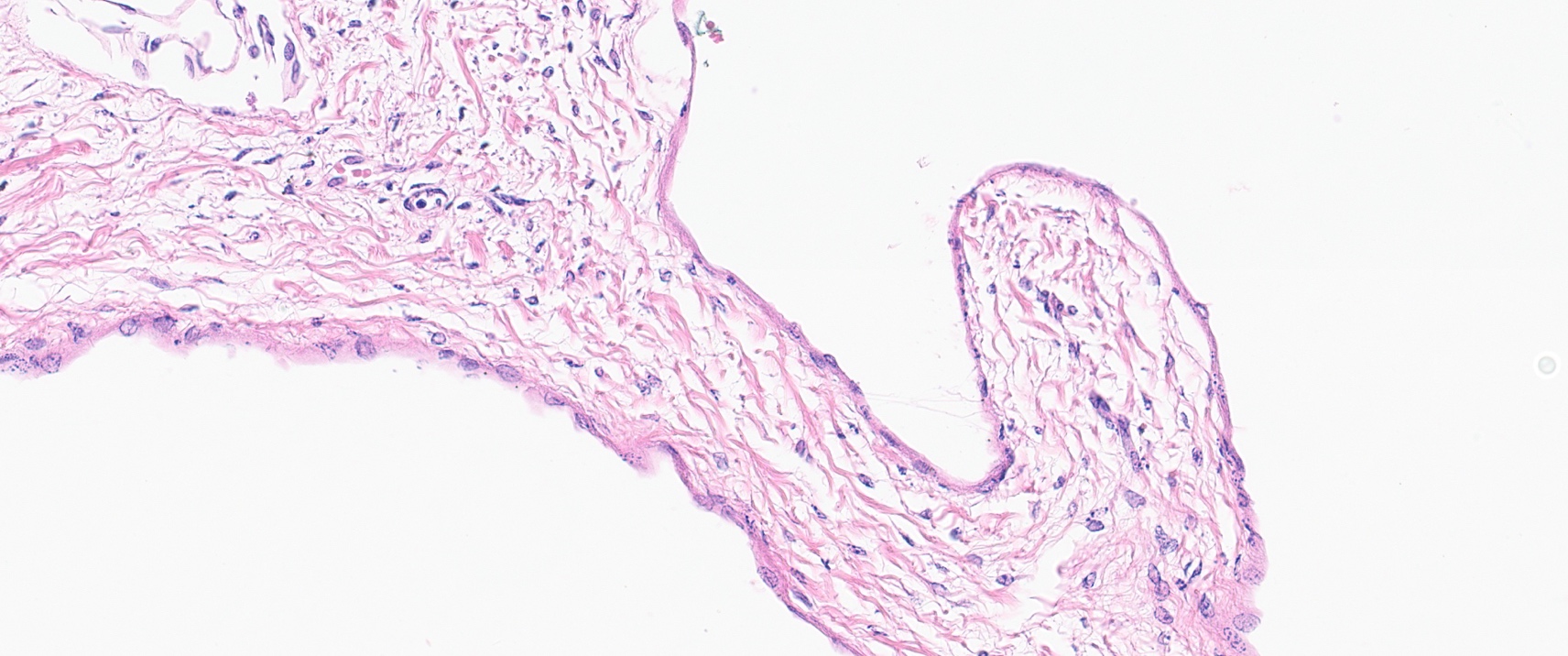


**D.** Fetal liver sections.

Up : Multiloculated cystic structure lined with simple squamous epithelium (x9.5).

Down : zoomed-in section of the cystic epithelium (x24).


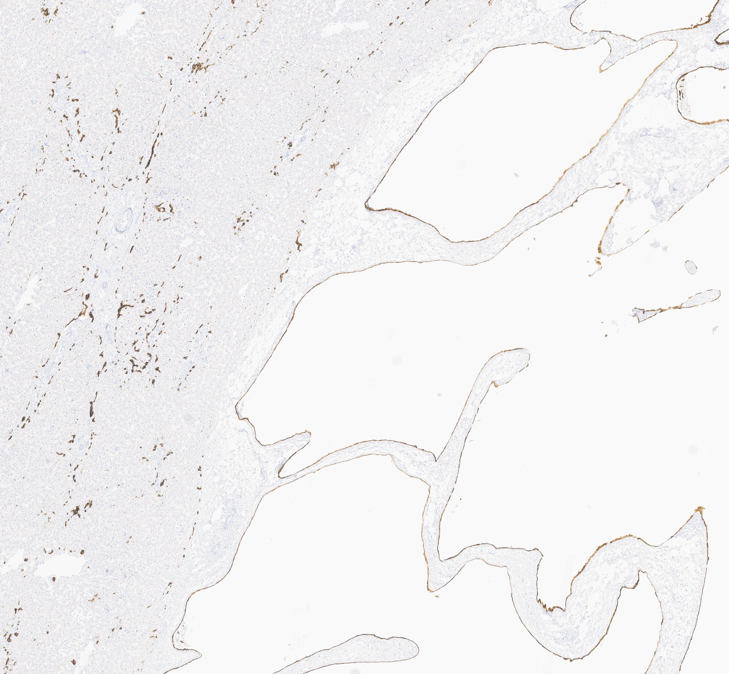


**E.** Immunohistochemistry showing CK7 expression by the epithelium, supporting biliary origin of the cyst.

**
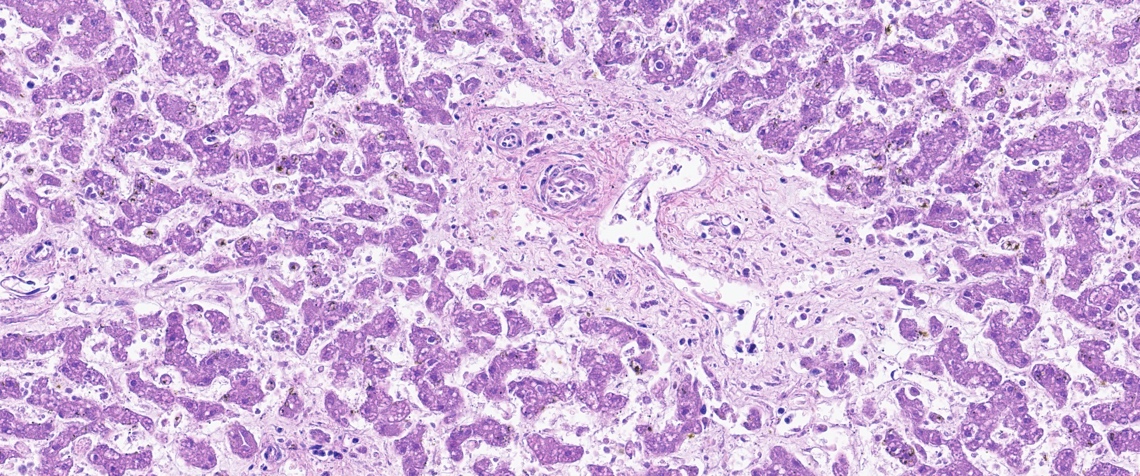
**
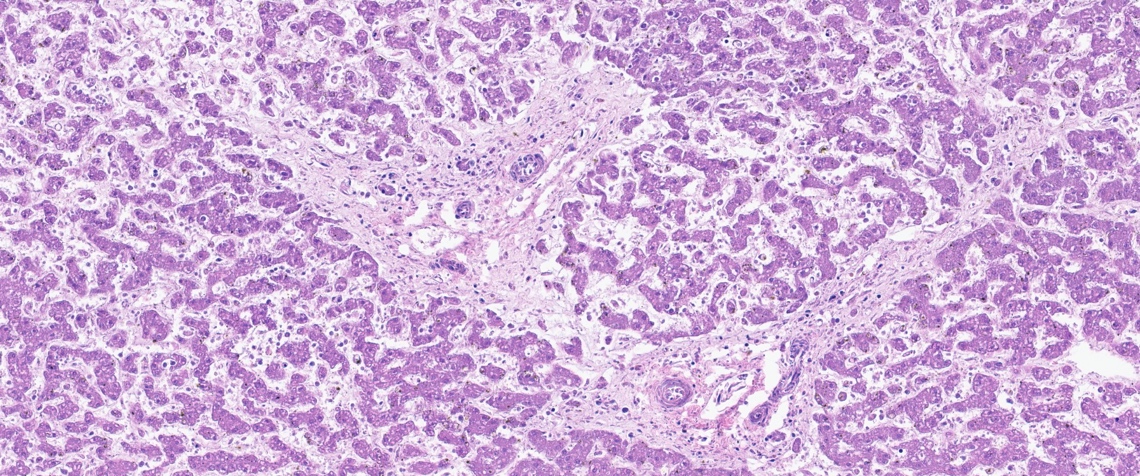

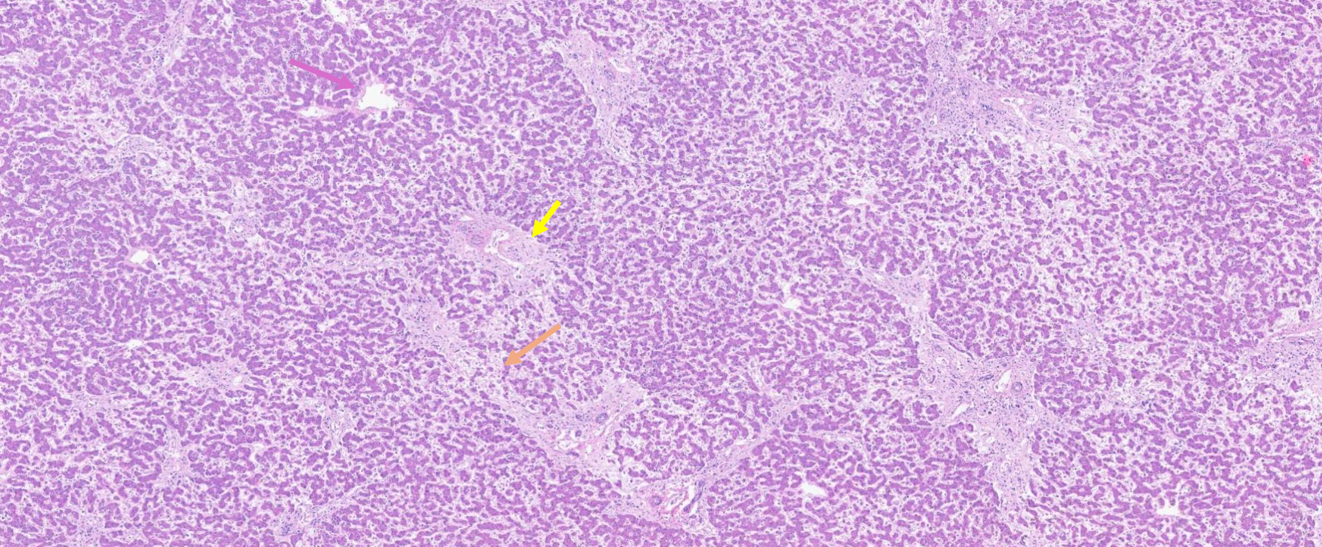


**F**. Fetal liver sections.

Up : Features of obliterative portal venopathy with fibro-edematous widening of portal tracts (yellow arrow) and porto-portal fibrous bridging (orange arrow). Some centrilobular veins are dilated with thickened walls, possibly due to right heart failure (pink arrows) (x4.8).

Bottom left : zoomed cross-section of the porto-porte bridges (x15).

Bottom right : zoomed cross-section of the fibro-edematous widening of portal tracts (x24).


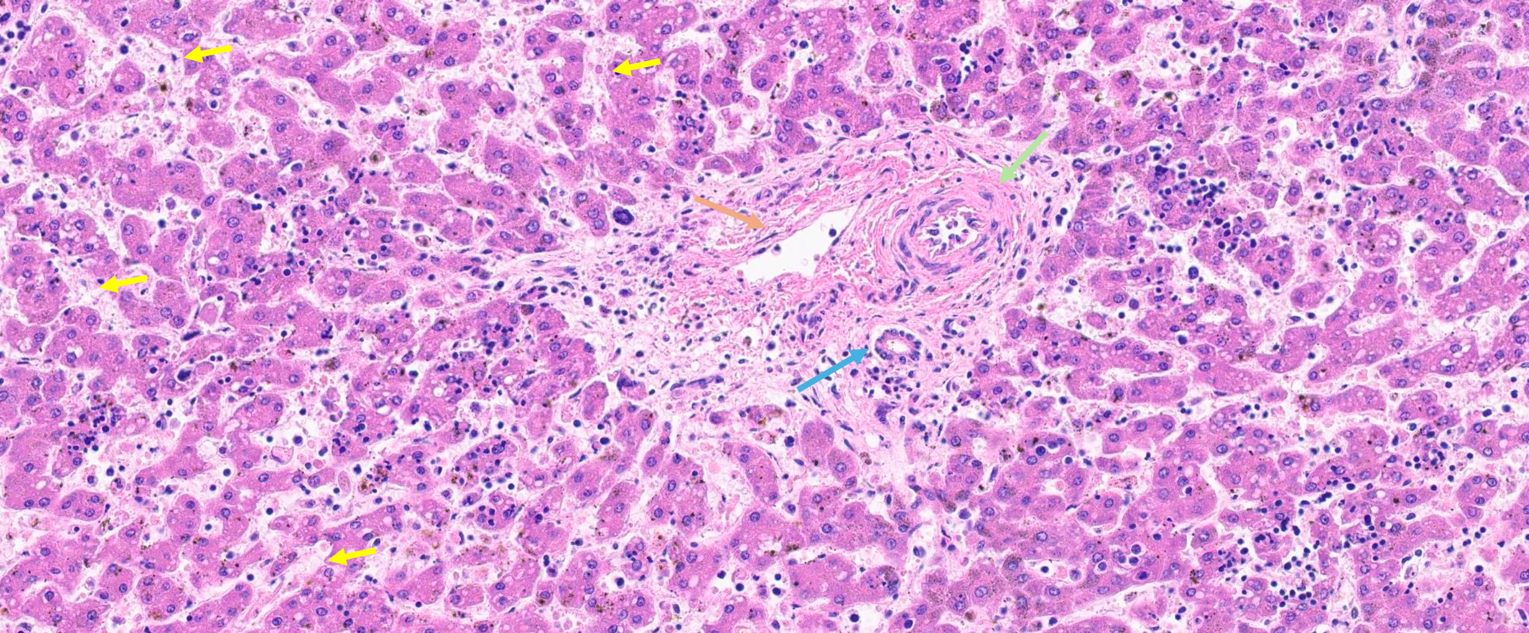


**G**. Fetal liver section centered on a portal tract (×19).

Hypoplastic portal vein branches (orange arrow), smaller than hepatic arterial branches (green arrow) or even absent in some sections. Blue arrow: bile duct. Mild sinusoidal dilatation in the parenchyma (yellow arrows).
