# Supplementary material for: Hypertrophic Cardiomyopathy as a Key Feature of MRAS‐Related Noonan Syndrome: New Case and Comprehensive Literature Review
Source: Prenat Diagn. 2026 Mar 22;46(4):581–8. doi: 10.1002/pd.70134 (PMC13070222; doi:10.1002/pd.70134)
Supplement: Supplementary file 2 — Supporting Information S2 [file PD-46-581-s002.docx]

**Appendix 2**

*Genetic Test Description :*

Exome sequencing from DNA extracted from fetal tissues. The technique used follows this process: from 50 ng of fragmented DNA, indexed libraries are prepared and hybridized with biotinylated probes from Twist Human Exome (37Mb). The samples are prepared according to the manufacturer’s recommendations. The library pools (multiplexed by 36 samples) are sequenced on the Illumina NextSeq2000 sequencer in paired-end mode (2x150 bp) on a FlowCell P3. Raw data (files in BCL format) are converted to FASTA format using Dragen software (Dragen BCLConvert v3.8.4) installed on the sequencer (Illumina). Sequences are analyzed following best practices recommended by the GATK Broad Institute, using two pipelines: Internal pipeline: BWA-MEM, GATK v3.6-44ge7d1cd2 ; SeqOne pipeline: v1.2, 2018. Variants are filtered based on coverage depth (DP>10), their frequency in GnomAD (<1%), their allelic frequency (>20%), their effect on the protein, their pathogenicity according to ClinVar and HGMD (as available at the time of analysis), and their compatibility with the phenotype and mode of inheritance. Only variants associated with a medical condition and considered clinically relevant to the index case’s phenotype, as described at the time of interpretation, were reported.

To ensure reliable clinical interpretation, coverage data are as follows : number of variants in RefSeq: 30,856 SNPs (reference value: approximately 30,000 SNPs) ; percentage of bases >10X: 98% (reference: >90-95%^1^) ; number of paired reads: 35 million (reference: >20 million).

Variants located in regions with coverage below 10X and/or difficult-to-characterize regions may not be detected. Intronic variations beyond +10 and -10 are not detected.

Interpretation : pathogenicity interpretation follows ACMG recommendations^2^ and those disseminated by the NGS-Diag network (March 2021).

Identity Verification: comparison of VCF data with a genotyping technique (AS-PCR) on 15 markers: confirmed.

References

1. Rehm HL, Bale SJ, Bayrak-Toydemir P, et al. ACMG clinical laboratory standards for next-generation sequencing. *Genet Med* 2013; 15: 733–747.

2. Richards S, Aziz N, Bale S, et al. Standards and guidelines for the interpretation of sequence variants: a joint consensus recommendation of the American College of Medical Genetics and Genomics and the Association for Molecular Pathology. *Genet Med* 2015; 17: 405–424.
